# Socio-economic and education related inequities in use of modern contraceptive in seven sub-regions in Uganda

**DOI:** 10.1186/s12913-023-09150-y

**Published:** 2023-02-28

**Authors:** Fredrick E Makumbi, Sarah Nabukeera, Nazarius Mbona Tumwesigye, Cissie Namanda, Lynn Atuyambe, Aggrey Mukose, Sarah Ssali, Ronald Ssenyonga, Ritah Tweheyo, Andrew Gidudu, Carole Sekimpi, Catherine Verde Hashim, Martha Nicholson, Peter Ddungu

**Affiliations:** 1 School of Public Health, Makerere University, New Mulago Hospital Complex, Mulago Hill Road, P.O. Box 7072, Kampala, Uganda; 2 School of Women and Gender studies, Makerere University, Makerere Hill, P.O. Box 7062, Kampala, Uganda; 3 Marie Stopes Uganda, Plot 1020 Rose Lane, Kisugu-Muyenga, P.o. Box 10431, Kampala, Uganda; 4 Marie Stopes International, 1 Conway Street, Fitzroy Square, London, W1T 6LP UK

**Keywords:** Equity, Family planning, Modern contraceptives

## Abstract

**Background:**

Advocacy for equity in health service utilization and access, including Family Planning (FP) continues to be a cornerstone in increasing universal health coverage. Inequities in Family planning are highlighted by the differences in reproductive health outcomes or in the distribution of resources among different population groups. In this study we examine inequities in use of modern contraceptives with respect to Socio-economic and Education dimensions in seven sub-regions in Uganda.

**Methods:**

The data were obtained from a baseline cross-sectional study in seven statistical regions where a program entitled “Reducing High Fertility Rates and Improving Sexual Reproductive Health Outcomes in Uganda, (RISE)” is implemented in Uganda. There was a total of 3,607 respondents, half of whom were women of reproductive age (15-49 years) and the other half men (18-54 years). Equity in family planning utilization was assessed by geography, wealth/economic and social-demographics. The use of modern family planning was measured as; using or not using modern FP. Concentration indices were used to measure the degree of Inequality in the use of modern contraceptives. Prevalence Ratios to compare use of modern FP were computed using modified Poisson regression run in STATA V15.

**Results:**

Three-quarters (75.6%) of the participants in rural areas were married compared to only 63% in the urban. Overall use of modern contraceptives was 34.2% [CI:30.9, 37.6], without significant variation by rural/urban settings. Women in the higher socio-economic status (SES) were more advantaged in use of modern contraceptives compared to lower SES women. The overall Erreygers Concentration Index, as a measure of inequity, was 0.172, *p*<0.001. Overall, inequity in use of modern contraceptives by education was highest in favor of women with higher education (ECI=0.146, *p*=0.0001), and the concentration of use of modern contraceptives in women with higher education was significant in the rural but not urban areas

**Conclusion:**

Inequities in the use of modern contraceptives still exist in favor of women with more education or higher socio-economic status, mainly in the rural settings. Focused programmatic interventions in rural settings should be delivered if universal Family Planning uptake is to be improved.

## Background

Advocacy for equity in health service utilization and access including Family Planning (FP) continues to be a cornerstone in increasing universal health coverage. On a global stage, the sustainable development goals (SDGs) were designed on the notion of leaving no one behind, with SDG3 calling for healthy lives for all ages, while SDG10 calling for the reduction of inequities within and between countries [1, 2]. Contraceptive use is a well-known intervention for improving women’s and children’s health as well as families’ wellbeing, by reducing the risk of maternal mortality and improving infant and child survival as a result of birth spacing [3]. These benefits of FP have been highlighted in health targets for SDG3, specifically SDG3.1 and SDG3.2 [1]. Given the enormous social, economic and health benefits of FP, there have been significant global efforts; including the recent Family Planning 2020 (FP2020) initiative that followed the London Summit on FP in 2012, to promote family planning [4].

While equality ensures FP resources are equally distributed among the different population sub-groups, equity ensures everyone has a fair opportunity to reach their reproductive health potential regardless of their social determinants of health. This is ensured with regard to availability, accessibility, acceptability, and quality of family planning services [5]. Besides, inequalities in family planning are unavoidable differences in FP access and utilization as a result of natural biological variations, for example male cannot be offered oral contraceptives and females cannot use vasectomy. On the other hand, inequities are unfair, undesirable, unnecessary and avoidable differences which infringes on human rights norms; for example the offering of specific FP methods to clients based on age or social status, by providers [6].

It is noteworthy to mention that equity in family planning does not mean that all groups use contraception inevitably at equal rates, but rather have the same access to information and services including available methods of contraception. In addition, individuals should be able to make decisions about their fertility and use of contraception and act on those decisions [6]. Equity for family planning entails distributing resources with respect to “need” of the sub-groups to improve health outcomes or to maintain health [7].

Inequities in Family planning care; are highlighted by the differences in maternal mortality, unwanted pregnancies or in the distribution of FP resources (unmet need, demand for FP satisfied and contraceptive prevalence rate) between different population groups [8–10]. Thus, although FP has been identified as a key accelerator of fertility declines that may lead to economic development and eventually the demographic dividend, inequities still exist between and within countries, especially in sub-Saharan Africa [11, 12]. In the Eastern and Southern Africa, there has been an increase in contraceptive use prevalence in some countries like Rwanda, yet others including Uganda have not reached their set targets [13]. The low mCPR has been attributed to several barriers to access FP services, including persistent social-cultural and economic challenges [14, 15]. Moreover, studies have highlighted a lower family planning use by sex, health status, refugee status and socioeconomic conditions, like income and education [2, 16, 17].

Improvement in reproductive health outcomes, including reduction in MMR and the total fertility rate, as well as an increase in contraceptive use have been observed in Uganda, between 2011 to 2016 [18, 19]. However, even with these improvements, Uganda’s TFR remains one of the highest in the world (5.4) [18], higher than the global average (2.4 ) and the East and Southern Africa region’s average (4.2) [20]. Moreover, use of modern contraception at 35% is lower than the country’s target of 50%, and the unmet need for FP at 28%, is nearly three times higher than the national target [21]. Likewise, key fertility determinants such as early sexual debut and first marriages are largely unchanged, and some data suggest disparities in the distribution and utilization of family planning services [19, 21].

A number of FP programs have been designed and implemented in Uganda to improve access to and utilization of FP services. However, the monitoring of contraceptive use based on strategic intervenable community and individual level characteristics like education and socioeconomic status, to enable further strengthening of the family planning services utilization and access remains limited [22, 23]. Real-time evidence from assessments with an equity lens is needed to facilitate targeted FP policy and programme decisions which will eliminate inequities in FP service access and utilization, improve the coverage and ultimately the reproductive health outcomes across the various population sub-groups in the country.

Previous studies have assessed inequities in the use of modern contraceptive majorly using large survey datasets like the demographic and Health Surveys (DHS) and Performance Monitoring for Accountability 2020 and Performance Monitoring for Action (PMA); however, the focus has been on the wealth and geography dimensions [11, 18, 21, 24]. Equality and universal coverage for FP have continuously or interchangeably been construed as equity. Although related, these concepts have different programmatic implication with inequity indicative of disproportionately, poor receipt or provision of health services based on community characteristics. We therefore overtly define inequity for family planning as; the unfair and avoidable disparities in the use of modern contraceptive across the different sub-populations, which infringe on human rights [25].

In this study, we examined inequities in use of modern contraceptives, based on key intervenable dimensions of wealth status and education compared by geography (type of residence, sub-region) or demographics (age, marital status, and disability status) in the seven study sub-regions of Uganda; to inform the RISE project and other FP stakeholders on the programmatic areas of focus that can reduce inequities in FP services and improve the health of women and children in Uganda.

## Methods

### Study design

Data for this analysis were accrued from a baseline cross-sectional study in seven statistical regions where the “Reducing High Fertility Rates and Improving Sexual Reproductive Health Outcomes in Uganda, (RISE)” project is implemented in Uganda. The seven sub-regions are based on the 2011 Uganda demographic and health survey as defined by the Uganda Bureau of Statistics (UBOS). The study regions were Central 1, Central 2, East-Central, Eastern, Karamoja, Western, and West Nile.

### Sample size and sampling

The sample size for the RISE baseline survey was a total of 3 607, half of them women of reproductive age (15-49 years) and the other half men (18-54 years). The sample size estimate was based on the following assumptions; intention to use FP of 62% as per PMA2020 (2016 survey) in the general population, desired margin of error $\delta$ at 4%, individual response rate at 80%, a household response rate at 80%, a design effect (DEFF) of 2 and resulting in a sample size of 1,767 each for male and female. This was adjusted for a a non-response rate of 2% as in the UDHS 2016 to result into 1,803 for each female and male sample separately. The male and female study sample was equally distributed across the 7 study regions. The final response rate was 74.7% (*n*=1803), resulting in 1346 females available for this analysis.

First, data collectors in each enumeration area (EA) mapped and listed all households. Where the number of households listed in an EA was less than 120, an adjacent EA was added (or annexed) for mapping, listing prior to sampling 60 households. Half (30) of the randomly selected households in each EA was assigned for eligible female respondents while the other half was for eligible male. Female eligibility was defined as being a usual household resident between 15 to 49 years of age, while male eligibility was usual household resident aged 18-54 years. Where more than one eligible participant was enumerated in a household, a systematic sampling was applied using a computer app installed on the smartphone to randomly select one member.

### Data collection

In each household, the details of name, index number, age and sex were entered into the pre-programmed listing form within the ODK online data collection software [26]. A random selection of one eligible participant per household was carried out using a code developed within the ODK’s programming enabling option. The selection of households followed a non-substitution policy and that means selected households were not replaced if respondents were unavailable [27]. Studies show that substitution leads to samples that do not match known population distributions [27]. Moreover, large surveys in the country by UBOS and PMA2020 also follow the non-substitution policy. At least 3 call backs were made at different times and days before declaring the respondents as unavailable for interview. Each research assistant interviewed a respondent of the same sex to improve quality of data.

### Measurements

For the purpose of this study, equity in family planning utilization was assessed along three dimensions i) Geography defined as rural/urban residence based on location of EA as assigned by national statistical office ii) wealth/economic, defined as lowest, lower, middle, higher and highest quintiles; measured by ownership/possession of household assets (Communication: cell or landline phone, computer, radio, TV; Transport: bicycle, motor cycle, car; Source of power: electricity, generator, solar, and materials for building/construction, ownership of the home, indoor bathroom, access to running water), and iii) Social-demographics that included marital status (Never married, married or divorced/widowed), highest level of education attained (None, Primary or Secondary and above) and age in completed years categorized as 15-19 years, 20-24 years, 30-39 years and 40-49 years.

Other socio demographics included: disability status as measured by the Washington Group (No-difficulty, some or a lot of difficulty) [28], Employment status (Unemployed, student or employed), and the 7 sub-regions (Western, Central-1, Central-2, East Central, Eastern, Karamoja and West Nile).

The intermediate variables mainly focused on the supply-side, defined as places used to get FP, measured by; access to a health facility/private or public, and the demand-side, defined as exposure of electronic and paper media FP messages and messages via village health teams (VHT), measured as; knows any FP method, knows a modern FP method, knows 3 FP methods, knows 5 FP methods, knows an organization/facilities that offer FP and heard about FP from any media in the past 2 weeks.

The primary outcome variable of interest for this analysis was use of modern family planning^1^, measured as; using or not using modern FP.

### Data management and statistical analysis

Although the large RISE project survey had both male and female, this analysis focuses only on eligible females aged 15-49.

#### Construction of analytical weights

All analyses were conducted with STATA software version 15 using the *surveyset* methodology that handles and accounts for the survey design. All the analyses were weighted, unless specified. The Uganda Bureau of statistics (UBOS) provided the EA selection weights. The EA selection weights were adjusted for probability of selecting a household per EA, eligible participants per sampled household and the non-response rate at EA level. The adjustments resulted into final weights that were used to weight the analysis.

The descriptive analyses were presented stratified by residence, rural/urban. Statistical weighting was done because of the multistage sampling approach, and the weights were scaled so as to sum the target population for the survey.

#### Analysis of socio-economic equity in utilization of modern family planning

##### Concentration curve

A graphical representation of how a health variable, use of modern contraceptives is distributed across population ordered characteristics such as education and wealth/ socioeconomic status measured as wealth-quintiles was done in this study. The cumulative proportion of a health variable is plotted on the y-axis against the cumulative proportion of the population/representative sample ordered/ranked by a characteristic; education or wealth/socioeconomic status. Wealth-quintiles or levels of education are ranked from the lowest to the highest on the x-axis. An equally distributed health variable across the wealth-quintiles/levels of education will result in the concentration curve with 45° line showing no inequality. However, if a health variable ranks with higher values among people in lower wealth-quintile or level of education, the concentration curve will lie above the line of equality. The further the curve lies from the line of equality, the greater the degree of Inequality in health. If, by contrast, the health variable ranks with lower values among people with lower wealth-quintile or level of education, the concentration curve will lie below the line of equality.

##### Concentration index

The concentration index provides a measure of the degree of Inequality in a health variable over the distribution of another variable. Concentration indices as a measure of inequality in one variable over the distribution of another [29] are a common choice for the measurement of socioeconomic-related health inequality [30]. We therefore used concentration index as a method of choice in this study to measure Inequity in use of modern contraceptives mCPR over the sample distribution of household wealth, and women’s education.  A comparison of these indices was made by residence (urban/rural), marital status, sub-regions and age (in years) to determine if these indices varied by the strata.

The standard version of the concentration index was derived from the concentration curve and represents twice the area between the concentration curve and the 45° line of equality. However, use or non-use of modern contraceptives was a bounded health variable, with binary indicators/outcome (0,1), we thus used the Erreygers Concentration Index, or ECI (proposed by Erreygers as modified version of the concentration index). The ECI satisfies the conditions that the absolute value of the index is the same regardless of whether the outcome used to assess Inequity is users or non-user of modern contraceptive (mirror property), and that the value of the index is invariant to any feasible positive linear transformation of the health variable (scale and translation invariance). The ECI is defined as:

$$ECI\left(h\right)=\frac{8}{{n}^{2}({b}_{h }-{a}_{h})}\sum_{i=1}^{n}{h}_{i}{R}_{i}$$

where h_*i*_ _is the health variable, use or non-use of modern contraceptives, *R*_*i*_ _is the fractional rank of woman *i* in the distribution of wealth-quintile status, *n* is the number of observations and *b*_h_ and *a*_h_ are the variables upper bound and lower bound, respectively^2^. The equation shows that the concentration index can be interpreted as a sum of weighted health levels, with the weights being determined by the wealth-quintile rank (or educational-level rank). The ECI is a measure of absolute Inequality for bounded variables. ECI values have a possible range from -1 to +1. It has a negative value when the health indicator is concentrated among the more disadvantaged (poor or less educated); and it has a positive value when the health indicator is concentrated among the more advantaged (rich or more educated). When there is no inequality, the ECI value is 0. The command *conindex* with the *erreygers* option in STATA v14.2 was used to calculate wealth and education-related Erreygers concentration indices. Similarly, a Wagstaff CI was used to arrive to the same conclusions.

The concentration index (CI) was used as index of health Inequality because it satisfies the minimum criteria for a health Inequality measures (i) it is reflective of the socioeconomic dimensions of health inequity, (ii) that it portrays the experience of the entire population, and (iii) it is sensitive to changes in the distribution of the population across socioeconomic groups. The generalized concentration index is appropriate in cases where absolute Inequality is of interest [31].

### Statistical modeling to determine factors associated with utilization of modern family planning methods

The final response rate was 74.7% (*n*=1803), resulting in 1346 females available for this analysis. Exploratory data analyses were conducted to generate descriptive statistics from the sample of the respondents especially the socio-demographic characteristics, and categorical variables were presented as weighted proportions.

A generalized linear model under *svyset* was used for the regression analyses with the use of modern contraceptive as the primary outcome coded “1” if participant was a current user and “0” if not a current user. The prevalence ratio (PR) was used as a measure of association instead of the odds ratio (ORs) because the primary outcome was common (greater than 10%), and this approach minimizes the overestimation of the association. The PR was obtained by using a “modified” Poisson regression model via a generalized linear model (GLM) with family as Poisson and a log link. The prevalence ratio (PR) compares the percent of the outcome between any two groups or levels of a variable. All PRs were estimated together with their corresponding 95% confidence intervals (CI).

A stepwise/logic analysis was conducted using the analytical conceptual framework starting with *equity in family planning utilization* dimensions. Geographical defined as rural/urban, economic based on wealth-quintile, and socio-demographic based on age- categorization as adolescent (15-19 years), young women (20-24 years), and older women grouped as 25-39 and 40-49 years, and marital status. Intermediate variables included supply (places ever used to get FP) and demand (knowledge and sources of information of FP methods).

Lastly, women individual characteristics (highest level of education attained and current employment status) were included. All these thematic areas were modeled separately for models 1 (unadjusted) and model 2 (adjusted within the thematic areas).

In order to identify factors independently associated with the outcome, only variables in model-2 that had *p*-value of 20% or less, or known confounders or factors from previous studies, were added to the final multivariable regression model-3 which has i) equity

dimensions, ii) individual and household level characteristics (when applicable) and iii) intermediate variables (supply and demand). All factors with statistical significance, *p*<0.05 were considered important. When we had two competing models, we used the Akaike's Information Criteria (AIC) to select the “best” model. Collinearity of the explanatory variables was assessed using the Stata variance inflation factor (VIF). All the key variables included in the final models did not violate the 10% threshold; e.g. age was selected over parity due to potential challenges of collinearity.

Three models were used to determine the factors associated with utilization of family planning. Model-2 statistics were used to inform model-3 to account for potential confounders that may have been missed in model-1. Some variables, like disability status, knowledge of i) any FP method, ii) any modern method and iii) 5 FP methods were collinear with 3+FP methods. We thus opted to consider only 3+FP methods.

Interaction of key predictors in the association with the primary outcome of interest was assessed. Assessing interaction terms is important because this can help identify and streamline targeted programmatic interventions geared towards improving service uptake or minimizing adverse outcomes. We therefore included an interaction term between age and marital status to enable us to further explain variation in use of modern contraceptives. The interaction term enabled us to better understand if associations between the use of modern contraceptives and marital status further vary by participants’ age categorization (adolescents, young or older age). Then, messages and actual service delivery and programs can be targeted. The statistical significance of the covariates and the interaction terms was determined using the Wald’s test (t*estparm* in Stata (version 15)).

The RISE survey has constructed sampling weights in the design of the survey. These weights were accounted for in order to obtain representative estimates, via use of the *svyset* commands in Stata. Use of generalized linear model (GLM) in the *surveyset* also accounts for clustering of observations at the lowest level, which was assumed at the EA level.

## Results

Table 1 shows the characteristics of participants stratified by rural/urban residence. Majority of the participants were from rural residence (73.1%). The percent of rural participants was highest in the Eastern (100%), followed by Western (92.6%), and lowest in Central-1 (35.8%). Overall, 29.4% of the participants were from the second highest wealth-quintile, followed by 24% in the lowest quintile. Only 3.3% of the rural were in the highest wealth-quintile compared to a third (34.2%) in the urban, while 11% of the urban were in the lowest wealth-quintile compared to 29.3% in the rural settings. Majority of the participants were aged 25-39 years (36.3%), higher in the urban (41.3%) compared to the rural (34.5%), while adolescents (15-19 years) were only 15.4%, higher in the rural (16%) compared to the urban (13.8%). Three-quarters (75.6%) of the participants in rural residency were married compared to only 63% in the urban, while self-reported disability (32.2% overall) tended to be higher in the rural (33.7%) relative to the urban (28%). Only a third of participants (32.1%) had at least secondary level of education, higher in the urban (55.1%) compared to the rural (23.5%), but both primary and no education were more common in the rural. Unemployment was higher in the urban (28.5%), compared to the rural (15.7%), while in the rural only 6.1% were students compared to 11.5% in the urban.

**Table 1:** Characteristics of participants stratified by rural/urban residence (weighted)

		**Residence**		**Overall**
	**Combined rural/urban**	**Rural**	**Urban**	**Total**
**Number**	**1359**	**73.1**	**26.9**	**100.0**
**Sub-regions**				**Row %**
Western	283	92.6	7.3	100.0
Central-2	171	79.5	20.4	100.0
Central-1	318	35.8	64.2	100.0
East-Central	189	73.5	26.4	100.0
Eastern	211	100.0	0.0	100.0
Karamoja	75	77.3	22.7	100.0
West Nile	112	66.2	33.7	100.0
**Characteristics**				**Column %**
**Wealth-quintile**				
Lowest	331	29.3	11.0	24.4
Lower	161	15.4	2.2	11.8
Middle	310	21.4	26.5	22.8
Higher	400	30.7	26.0	29.4
Highest	157	3.3	34.2	11.6
**Age-categories (years)**				
15/19	210	16.0	13.8	15.4
20/24	313	21.6	26.9	23.0
25/39	494	34.5	41.3	36.3
40/49	343	27.9	17.9	25.2
**Marital status**				
Never-Married	234	13.6	27.2	17.2
Married	981	75.6	63.0	72.2
Divorce/Separated/widowed	143	11.0	9.5	10.6
**Self-reported disability**				
None	921	66.3	71.8	67.8
At least one	438	33.7	28.2	32.2
**Level of education attained**				
None	197	16.9	8.0	14.5
Primary	726	59.6	36.7	53.4
Secondary or higher	436	23.5	55.1	32.1
**Current employment status**				
Unemployed	260	15.7	28.5	19.1
Student	102	6.1	11.5	7.5
Employed	996	78.2	60.0	73.3

### Inequity in use of modern contraceptivesby wealth

**Fig. 1:**
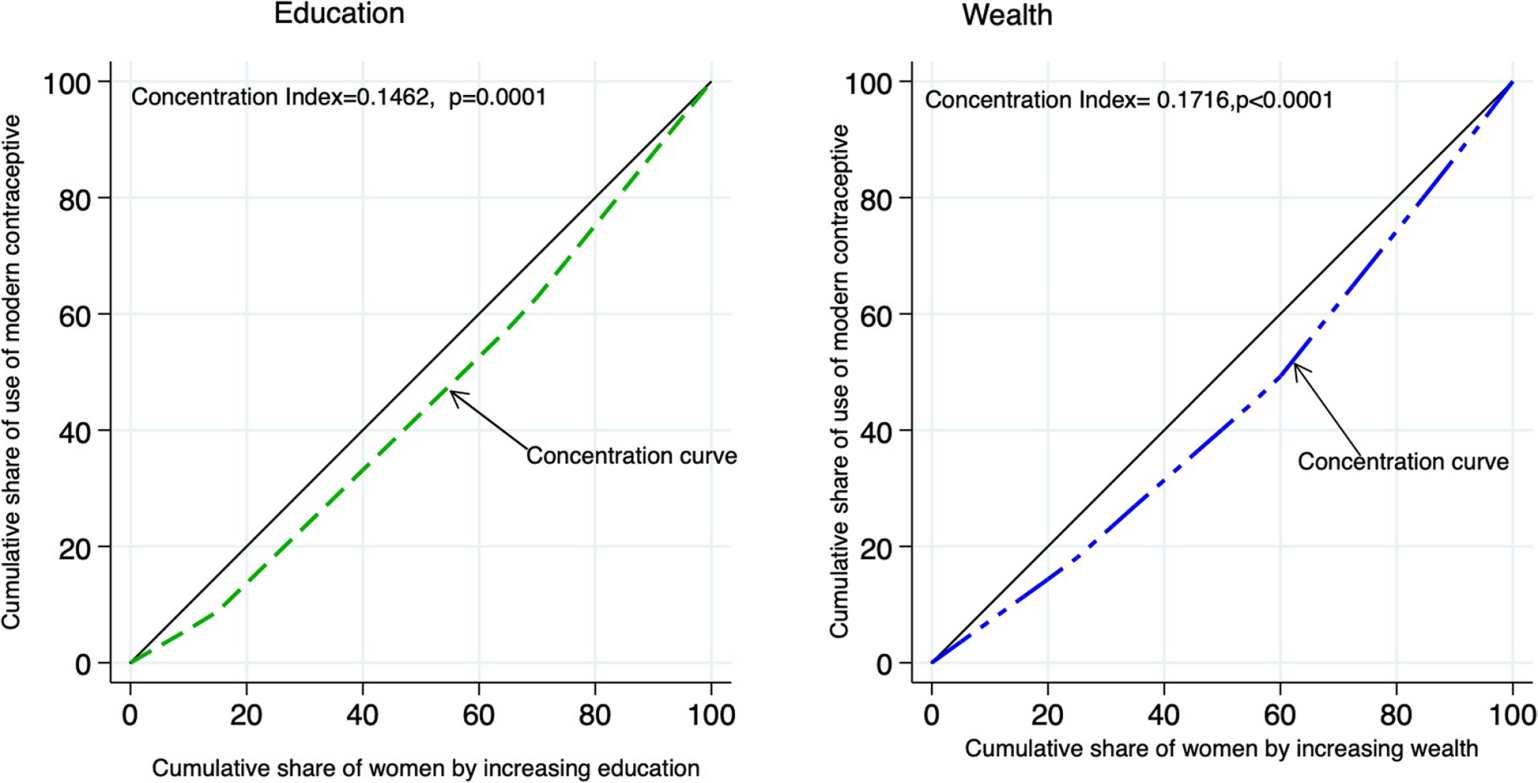
Concentration curves for utilization of modern contraceptive in 7 sub-regions in Uganda

Figure 1 shows the concentration curves for utilization of modern contraceptive in 7 sub-regions in Uganda, while Table 2 shows the Erreygers Concentration index (ECI) of Inequity in use of modern contraceptives by wealth status and level of education compared for: the type of residence, sub-region, age (years), disability status and, marital status. Overall, concentration of use of modern contraceptives was among the wealthiest (higher/highest wealth quintile) women, with the Erreygers Concentration Index, ECI=0.172, *p*<0.001.

**Table 2:** Erreygers Concentration index (ECI) of Inequity in use of modern contraceptives by wealth and Education compared by the levels of residence, age (years), sub-region and marital status

	**Wealth-Quintile**				**Education level**		
	**N**	**Absolute ECI**	**SE**	***p*-value**	**Absolute ECI**	**SE**	***p*-value**
**Overall**	1341	0.172	0.032	*<0.001*	0.146	0.035	*0.0001*
**Residence**							
Rural	1071	0.145	0.041	*0.0009*	0.137	0.041	*0.0015*
Urban	270	0.213	0.053	*0.0018*	0.137	0.089	0.1493
Difference between 2 groups		0.067	0.067	0.3159	0.000	0.098	0.9983
Difference in groups				0.4303			0.935
**Sub-region**							
Western	225	0.150	0.083	0.1054	0.082	0.048	0.118
Central-2	197	0.095	0.065	0.1792	0.118	0.070	0.127
Central-1	215	***0.143***	***0.063***	***0.0466***	0.027	0.077	0.7327
East-Central	194	0.157	0.070	0.0549	***0.238***	***0.057***	***0.0032***
Eastern	221	0.125	0.061	0.0672	0.145	0.103	0.1903
Karamoja	178	***-0.002***	***0.001***	***0.0075***	0.019	0.024	0.4548
West Nile	111	-0.007	0.067	0.9240	***0.191***	***0.033***	***0.0043***
Difference in groups				0.7043			0.1439
**Age (years)**							
15-19	199	0.071	0.063	0.2596	0.015	0.059	0.8067
20-24	306	0.238	0.062	*0.0003*	0.1619	0.062	*0.0107*
25-39	483	0.165	0.055	*0.0035*	0.134	0.057	*0.0228*
40-49	353	0.178	0.057	0.0029	0.211	0.064	*0.0015*
Difference in groups				*0.0054*			0.0049
**Marital status**							
Never-married	206	0.155	0.061	*0.0144*	0.145	0.057	*0.0133*
Married	999	0.227	0.040	*<0.001*	0.197	0.040	*<0.001*
Divorced/Widowed	136	0.050	0.097	0.609	0.071	0.080	0.3804
Difference in groups				0.1023			0.2007
**Disability status**							
No-difficulty	914	0.194	0.041	<0.001	0.1401	0.0393	0.0007
Some or a lot difficulty	427	0.105	0.057	0.0704	0.134	0.0532	0.0146
Difference in groups				0.0592			0.4001

This Inequity is more apparent in the urban ECI=0.213 (0.053, *p*=0.0018) compared to rural settings, ECI=0.145 (0.041, *p*=0.0009), but the difference by residence ECI=0.067(0.067) was not statistically significant (*p*=0.3159). We also observed the concentration of use of modern contraceptives in the wealthiest women as being significant in sub-region of Central-1, ECI=0.143(0.063, *p*=0.0466); women aged 20-24 years, ECI=0.238(0.062, *p*<0.001), 25-39 years, ECI=0.165 (0.055, *p*<0.01) and 40-49 years, ECI=0.178(0.057, *p*<0.01); the never-married, ECI=0.155(0.061, *p*=0.0144) and the married, ECI=0.227(0.040, *p*<0.001). However, in Karamoja the use of modern contraceptives was concentrated in the poorest women, ECI=-0.002(0.001, *p*=0.0075).

### Inequity in use of modern contraceptives by education level

The overall Inequity in use of modern contraceptives by education was highest in favor of women with higher education, ECI=0.146(0.035, *p*=0.0001) but this did not vary by residence (urban/rural), *p*=0.9983. We also observed the concentration in use of modern contraceptives in the women with higher education as being significant in sub-region of East-Central, ECI=0.238 (0.057, *p*<0.0032) and in West-Nile ECI=0.191 (0.033, *p*=0.0043); women aged 20-24 years, ECI=0.162 (0.062, *p*=0.01017), 25-39 years, ECI=0.134 (0.057, *p*=0.0228) and 40-49 years, ECI=0.211 (0.064, *p*=0.0015); never-married, ECI=0.145(0.057, *p*=0.0133) and married, ECI=0.197(0.040, *p*<0.001), as shown in Table 2.

### Factors associated with use of modern contraceptives

Table 3 shows use modern contraceptive prevalence by measures of equity, individual and intermediate variables (weighted totals and use of modern contraceptives). The overall of use of contraceptives was 34.2% [CI:30.9, 37.6], higher in the urban compared to rural settings but the difference was not statistically significant (*p*=0.2455). The use of modern contraceptives significantly varied by sub-regions, highest in East-Central, 40.3% [CI:33.38, 47.61] and lowest in Karamoja, 7.4% [CI:4.4, 12.22], *p*<0.001.

**Table 3:** Modern contraceptive prevalence by equity, Individual, and intermediate variables (weight totals and use of modern contraceptives)

	**Number**	**Use of modern contraceptives, %**	**95%CI**	
Overall	**1359**	**34.2**	**30.9**	**37.6**
**Geographical residence**				
Rural	994	32.9	28.90	37.22
Urban	365	37.6	31.47	44.16
**Sub-regions (7)**				
Western	283	33.9	26.98	41.64
Central-2	171	37.9	30.95	45.33
Central-1	318	36.6	30.49	43.23
East-Central	189	40.3	33.38	47.61
Eastern	211	40.0	29.76	51.29
Karamoja	74.4	7.4	4.40	12.22
West Nile	112	18.6	14.14	24.15
**Wealth quintile**				
Lowest	443	24.5	19.75	30.03
Lower	161	30.6	23.06	39.27
Middle	310	29.9	24.87	35.43
Higher	400	42.7	36.27	49.42
Highest	157	45.0	36.99	53.21
**Market segmentations**				
Rural_poor	444	27.4	2.22	23.15
Urban poor	48	18.6	7.77	7.54
Middle-income	310	29.9	2.64	24.87
Rural wealth	337	42.8	3.66	35.67
Urban wealth	219	44.2	3.21	37.92
**Age category**				
15/19	210	14.4	9.5	21.1
20/24	313	41.2	34.9	47.8
25-39	494	42.7	37.7	47.8
40/49	343	27.6	21.9	34.2
**Marital status**				
Never-Marry	234	22.9	15.4	32.6
Married	981	37.7	33.4	42.1
Divorce/widow	143	28.8	20.3	39.2
**Disability status**				
No-difficulty	921	36.3	32.44	40.27
Some or a lot difficulty	438	29.8	25.12	34.95
**Highest education attained**				
None	197	19.7	14.36	26.32
Primary	726	33.3	29.52	37.22
Sec+	436	42.3	36.10	48.73
**Employment status**				
Unemployed	260	35.6	28.33	43.59
Student	102	18.9	9.74	33.59
Employed	996	35.4	30.97	40.08
**Demand/knowledge**				
**Knows any FP method**				
No	81	2.3	0.46	10.68
Yes	1278	36.2	32.63	39.95
**Knows any modern**				
No	93	3.1	0.90	9.89
Yes	1266	36.5	32.86	40.21
**Knows 3 FP methods**				
<3	330	23.4	18.25	29.59
3+	1028	37.6	33.58	41.86
**Knows 5 FP methods**				
<5	910	30.2	26.82	33.84
5+	448	42.2	36.37	48.33
**Knows FP organizations/facilities**				
None	105	11.3	5.50	21.63
Only-one	640	30.3	25.71	35.22
At least2	614	42.2	37.31	47.22
**Any FP media heard in past 2wks**				
None	503	30.3	26.2	34.8
Only-one	612	34.9	29.7	40.6
At least2	244	40.2	34.0	46.7
**Supply**				
**Places ever used to get FP**				
None	488	9.0	5.60	14.26
NGOs/Hospitals/HC	871	48.3	43.74	52.83

Beyond residence and sub-region, use of modern contraceptives significantly varied by: wealth-highest-quintile, 45%[CI: 36.99, 53.21] versus lowest-quintile 24.5%[CI:19.75, 30.03], *p*<0.001, high in women aged 20-24 years, 41.2% [CI: 34.9, 47.8], those aged 25-39 years, 42.7% [CI: 37.7,47.8] and lowest in the adolescents (15-19 years), 14.4%[CI: 9.52, 21.14] *p*<0.001, and among the married 37.7% [CI: 33.4, 42.1] compared to the never-married, 22.9%[CI: 15.4, 32.6], *p*=0.0103, and in women with no reported form of disability, 36.3%[CI:32.44, 40.27] compared to 29.8%[CI:25.12, 34.9], *p*=0.0337 for women reporting at least one form of disability.

Table 4 shows the unadjusted and adjusted prevalence ratios (PR) and 95 % confidence intervals by measures of inequity, individual and intermediate variables. Three models were generated with model-1 having unadjusted PR of use of modern contraceptives, while model-2 with adjusted PR of either equity dimensions alone, individual characteristics or demand/supply variables alone. Lastly model-3 provides the final adjusted prevalence ratios of use of modern contraceptives for all significant variables in model-2 and the geographical rural/urban variable included irrespective of its statistical significance.

**Table 4:** Modern contraceptive prevalence, unadjusted and adjusted prevalence ratio (95%CI)

		**Prevalence Ratio (PR)**		
		**Model 1**	**Model 2**	**Model 3**
	**Use of modern contraceptives, %**	**Unadjusted 95%CI**	**Adjusted 95%CI**	**Adjusted 95%CI**
Overall	**34.2 [31.0-37.7]**			
**Geographical residence**				
Rural	32.9	1.0	1.0	1.0
Urban	37.6	1.14[0.91-1.43]	1.08 [0.89-1.32]	1.04[0.89-1.23]
**Sub-regions (10)**				
Western	33.9	1.0	1.0	1.0
Central-2	37.9	1.12[0.84-1.49]	1.05[0.80-1.38]	1.00 [0.80-1.24]
Central-1	36.6	1.08[0.82-1.43]	0.95[0.69-1.31]	1.00[0.81-1.22]
East-Central	40.3	1.19 [0.90-1.57	1.20 [0.95-1.52]	1.22 [1.00-1.49]
Eastern	40.0	1.18[0.83-1.67]	1.36[0.98-1.90]	1.27 [0.98-1.65]
Karamoja	7.4	0.22[0.13-0.38]	0.24[0.13-0.45]	0.41[0.26-0.65]
West Nile	18.6	0.55[0.39-0.78]	0.60[0.38-0.97]	0.71[0.57-0.89]
**Wealth quintile**				
Lowest	24.5	1.0	1.0	1.0
Lower	30.6	1.25[0.87-1.79]	0.99[0.68-1.46]	1.01[0.70-1.45]
Middle	29.9	1.22 [0.94-1.58]	0.96[0.72-1.29]	0.95[0.72-1.24]
Higher	42.7	1.74[1.36-2.23]	1.41[1.11-1.80]	1.21[0.95-1.53]
Highest	45.0	1.83[1.39-2.42]	1.56[1.15-2.11]	1.19[0.88-1.60]
**Age category**				
15/19	14.4	1.0	1.0	1.0
20/24	41.2	2.87[1.80-4.57]	*6.77[2.30-19.88]*	3.81[1.41-10.27]
25/39	42.7	2.97[1.96-3.75]	*3.75[1.24-11.35]*	2.11[0.73-6.07]
40/49	27.6	1.92[1.27-2.91]	3.22[0.85-12.21]	2.12 [0.60-7.43]
**Marital status**				
Never-Marry	22.9	1.0	1.0	1.0
Married	37.7	1.64[1.10-2.46]	3.22[1.15-9.05]	2.02 [0.72-5.67]
Divorce/widow	28.8	1.26[0.75-2.11]	8.92 [3.40-23.39]	6.91[2.91-16.43]
**Interaction term (Age and Marital)**				
20/24#Married			0.26[0.07-0.94]	0.38 [0.11-1.27]
20/24#Divorce/widow			0.06[0.01-0.32]	0.09 [0.02-0.39]
25/39#Married			0.55[0.15-1.93]	0.78 [0.23-2.65]
25/39#Divorce/widow			0.17[0.05-0.60]	0.19 [0.06-0.59]
40/49#Married			0.43[0.10-1.86]	0.56 [0.14-2.30]
40/49#Divorce/widow			0.10[0.02-0.46]	0.11 [0.03-0.43]
**Disability status**				
No-difficulty	36.3	1.0		
Some or a lot difficulty	29.8	0.82[0.68-0.99]		
**Highest education attained**				
None	19.7	1.0	1.0	1.0
Primary	33.3	1.69[1.27-2.26]	1.72[1.29-2.31]	1.25 [0.97-1.59]
Sec+	42.3	2.15[1.54-3.01]	2.39[1.71-3.34]	1.54[1.16-2.04]
**Employment status**				
Unemployed	35.6	1.0	1.0	
Student	18.9	0.53[0.26-1.08]	0.45[0.23-0.88]	
Employed	35.4	0.99[0.75-1.31]	1.04[0.81-1.34]	
**Demand/knowledge**				
**Knows any FP method**				
No	2.3	1.0		
Yes	36.2	15.80[3.19-78.17]		
**Knows any modern**				
No	3.1	1.0		
Yes	36.5	11.93[3.54-40.22]		
**Knows 3 FP methods**				
<3	30.2	1.0	1.0	
3+	42.2	1.61[1.23-2.09]	1.11[0.86-1.43]	
**Knows 5 FP methods**				
<5	23.4	1.0		
5+	37.6	1.57[1.19-1.64]		
**Knows FP organizations/facilities**				
None	11.3	1.0	1.0	
Only-one	30.3	2.69[1.33-5.45]	1.19[0.69-2.03]	
At least two	42.2	3.75[1.87-7.52]	1.50 [0.89-2.53]	
**Heard about FP from any media in past 2wks (sources)**				
None	30.3	1.0	1.0	
Only-one	34.9	1.15[0.95-1.40]	1.05[0.88-1.27]	
At least two	40.2	1.33[1.06-1.66]	1.10[0.87-1.38]	
**Supply**				
**Places ever used to get FP**				
None	9.0	1.0	1.0	1.0
NGOs/Hospitals/HC	48.3	5.34[3.24-8.79]	4.92[2.91-8.30]	4.24[2.54-7.06]

Knowledge of at least three methods of FP, or facilities/organizations providing FP and heard of FP from at least two media sources and places ever used to get FP services were significantly associated with higher use of modern contraceptives.

**Fig. 2:**
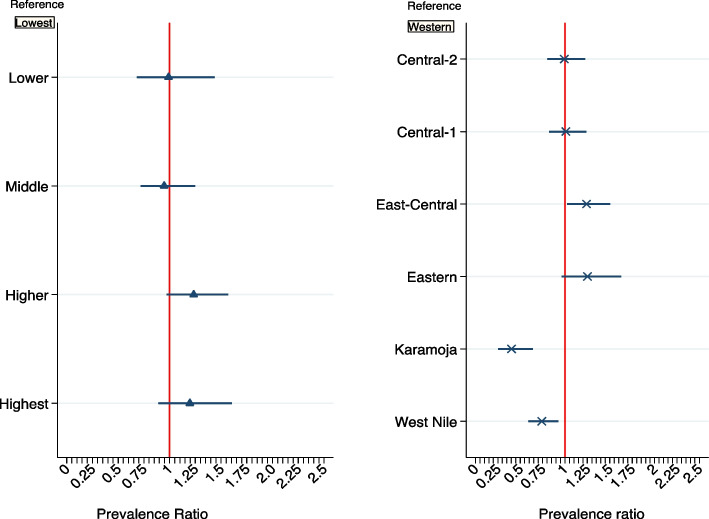
Adjusted prevalence ratios (adj. PR) comparing modern contraceptive rate of each Wealth quintiles against the lowest, and all regions against Western

In the final model 3, use of modern contraceptives was similar between rural/urban settings. In comparison to Western sub-region, use of modern contraceptives was significantly lower in Karamoja, adj. PR=0.41[CI:0.26, 0.65] and West-Nile, adj. PR=0.71 [CI: 0.57, 0.89] but significantly higher in East-Central, adj. PR=1.22 [CI:1.00, 1.49] and marginal in Eastern, adj. PR=1.27 [CI:0.98, 1.65] as shown in Fig. 2.

As shown in Fig. 2, women in the highest two-wealth quintiles had a 20% higher use of modern contraceptives compared to the lowest quintile, but this was not statistically significant in the final model (3). For the interaction term in Table 4, the ratio of use of modern contraceptives for the 20-24, 25-39 and 40-49 year olds compared to the 15-19 year olds, was significantly lower among the divorced/widowed compared to the never-married women, 0.09[CI: 0.02,0.39] *p*=0.002, 0.19[CI: 0.06,0.59] *p*=0.005 and 0.11[CI: 0.03,0.43] *p*=0.002, respectively.

Women with the secondary or higher level education compared to those with no-education had a 54% higher use of modern contraceptives, adj. PR=1.54 [CI:1.16, 2.04] while women with primary education had 25% higher use of modern contraceptives compared to no-education but this difference was not statistically significant, adj. PR=1.25[CI:0.97, 1.59]. Also, prior receipt and use of FP services was associated with current use of modern contraceptives, adj. PR=4.24 [CI: 2.54, 7.06].

## Discussion

This study shows that use of modern contraceptives is disproportionately concentrated among wealthier and more educated women in Uganda, especially in favor of women in the urban areas, those aged 20 years and above, and with regional variations. We also found that use of modern contraceptives was higher among women who have ever used NGOs/Hospitals/Health Centers to get FP services and commodities, secondary or higher level of education, and in the East-Central region.

In this study, the concentration in use of modern contraceptives in women with higher education is significant in the rural but not urban areas, whereas, that of wealthier women was significant for both rural and urban areas. In the same way, prior evidence cites that; in sub-Saharan Africa, family planning programs may initially have reached out to better-off clients, especially in urban areas, and now to promote equity, programs ought to emphasize efforts that increase FP access to those in rural and peri-urban areas [32]. Our findings support this, and further suggest that; family planning programs need to reduce socioeconomic and education related inequities in use of modern contraceptives that favor the wealthier or more educated, by targeting FP services to the socioeconomically disadvantaged women, or women with less education especially in the rural areas.

We also observed the concentration in use of modern contraceptives among the wealthiest women and those with higher education as being significant, for women aged 20 years and above compared to adolescents,15-19-year-old. Studies have shown that; for sub-groups like the young, almost half of the women in need were not using an effective family planning method [11, 33]. This finding may partially explain the persistent teenage pregnancies, the increasing unintended pregnancies and high maternal mortality in Uganda [19, 21]. A study on barriers to modern FP uptake among young women in Tanzania indicated myths and misconceptions, and fear of side effects as the core barriers, as well as unavailability of the preferred method and absence of the trained personnel for the FP method, although the intimate partner or closest friends were significant decision influencers on contraceptive use, [34, 35]. It is imperative thus, for FP programmers to consider young people customized interventions, unique to their needs and preferences to improve contraceptive use and ultimately reduce the associated poor reproductive health outcomes in the country.

A study in Nigeria showed a higher uptake of any FP method among the un-married compared to the divorced/widowed women. Similarly, this study also suggests that FP interventions be geared towards the 20-24, 25-39 and 40-49 year-olds for the divorces/ widowed as compared to 15-19yr, while among the never married, focus may be geared towards the 15-19 year-olds. This emphasizes the importance of evidently understanding and taking in to consideration each sub-groups’ context and particularities while planning FP programs [24], in order to reduce the inequities for FP in Uganda.

In this study, there was substantially a higher concentration in use of modern contraceptives among the wealthiest and women with higher education by sub-regions, indicating regional inequities. Similarly, a previous study cited that, inequities in Uganda were widely spread across country’s 15 regions and virtually every region struggled to provide equitable access to family planning information and services [19, 33].

These findings bolster the importance of addressing inequities for FP by the FP programmers in Uganda, taking into account the dimension of regions, if the impacts of FP interventions are to be attained.

Previous studies have cited absence of equitable reproductive health access among the disabled [36–38] and poorer reproductive health outcomes for this population sub-group [36]. Likewise, in this study, we found that use of modern contraceptives was higher in women with no reported form of disability compared to those reporting at least one form disability. Family planning information and services should be physically and geographically accessible, and affordable, for all [6], if we are to achieve equity. Besides, information should be evidence based and widely available in forms consistent with people’s needs [39]. It is essential therefore, for FP programs in Uganda to generate context-specific evidence on why this sub-group may be having low FP uptake compared to their counterparts [9]. With a deeper understanding of such inequities, informed effective targeted interventions will be implemented, to increase FP-access and improve the related reproductive outcomes among the disabled people.

In this study, knowledge of at least three methods of FP, or facilities/organizations providing FP and heard of FP from at least two media sources and places used to ever get FP services were significantly associated with higher use of modern contraceptives. This finding is in agreement with previous literature, which has cited that; effective counseling, and community-based behavioral-change communication programs aimed at improving the perceptions of women to bridge knowledge gaps about contraceptive methods and to changing deep-seated negative beliefs related to contraceptive use [40, 41], were needed to increase modern contraceptive use.

However, such behavioural change interventions also have to be tailored to the sub-groups’ unique information needs, as a strategy to effectively address the inequities in FP utilization and ultimately achieve universal coverage for FP in Uganda.

Although the Uganda Ministry of Health through the various FP programs has undertaken some of the evidence-based approaches including: enhancing mobile outreaches, village health centers, social franchising, which are especially successful in reaching rural and poorer clients through community health workers [28, 42, 43], there is need to modify, strengthen and scale-up these, informed by evidence, targeting the disfavored sub-groups; while fundamentally ensuring the quality of FP services especially in public health facilities to correct misconceptions about modern methods among rural women [8].

Furthermore, availing a broad range contraceptives through pharmacies and drug shops, to harness FP-self-care interventions [20, 28], while considering particular women’s fertility intentions and the sub-groups’ FP needs [12], could be a possible avenue to close such equity gaps in FP programming.

For financial barriers to FP access, the utility of vouchers and use of the ‘‘total market approach,’’ which encourages the better-off to use private-sector services so as to free up public-sector services for less well-off clients, may be optimized by FP-programmers [28, 29, 32], while coordinating the public and private sectors to streamline and maximize the benefits of the services in the country [20]. More to that, there is need to design, implement and monitor adolescent-customized interventions, that address not only the supply side but also the social norms that may deter contraception uptake for this age-group [11, 28], implying that FP campaigns should focus beyond the individual level and health facility system factors to address such inequities for FP in Uganda.

We used cross-sectional survey data, which can only show associations rather than infer causality between the equity dimensions and the use of modern contraceptives. Furthermore, for this study we did not consider other important factors like culture or partner-related characteristics that could influence women’s use of contraception, as well as the low prevalence of disability that limits the precision of the inequity estimate in this group. But from our findings, we confidently state that the need for contraception is not being fully addressed among all the sub-groups in Uganda, disfavoring the vulnerable women.

Additionally, this study may serve as a basis for future studies that may set out to assess inequities for FP; since measuring inequities using appropriate indicators, identifying who/where to intervene effectively, recognizing the underlying multifaceted contributors and effective monitoring, are critical; to promote FP uptake opportunities for all people regardless of their social background [30], broadly, equitably improving the health and health outcomes across the various population sub-groups in Uganda.

### Strengths and limitations

To our knowledge, this is one of a few studies that have provided evidence on inequities in a family planning indicator, use of modern contraceptives, and is based on a demographic health survey design. The self-reported outcome variable (use of modern contraceptives) might have influences of social desirable responses because the study communities are receiving FP intervention from the RISE project. However, this was minimized by use of well trained and experienced research assistants, and the findings of use of modern contraceptives are consistent with recent national level surveys..

### Recommendations

FP implementing partners need to target FP services (information, commodities and supplies) to young girls and women with no or low education levels or the socioeconomically disadvantaged across geographies and marital status. Increasing community access to a broad range of contraceptives through outreaches and subsidized self-care through pharmacies and drug shops could improve uptake among the socio-economically disadvantaged women. Broadly, these interventions may close/further minimize the inequity gaps in FP programming.

Further analysis to determine to what degree the inequities are due to FP demand generation, or supplies/service provision to the disadvantage sub-population should be conducted. This will enable subgroup context specific FP programs interventions/strategies to minimize especially the education and socio-economic related inequities in use of modern contraceptives.

FP implementing partners may need to engage with government programs as such as operation wealth creation (OWC) and local Savings and Credit Cooperative Societies (SACCOS) to strengthen FP within this government program and to also ensure that women are involved in the economic development programs. Enhanced socio-economic wellbeing will then support close the economic gaps and thus minimize socio-economic inequity in use of modern contraceptives.

FP implementation partners may need to collaboratively work with government institutions that promote and ensure universal primary and secondary education so that all girls of school going age can be encouraged to enroll and be retained into formal education as per the UPE/USE policy, because of the subsequent long-term benefits of education to FP programing.

## Conclusion

Socioeconomic and education related inequities in the use of modern contraceptives substantially exist among the different population sub-groups in Uganda, especially disfavoring adolescents and rural women. Thus, targeted interventions need to be devised to address the unique FP-needs of these subgroups, if universal FP coverage is to be attained.

## Data Availability

The dataset generated and analyzed during the study are not publicly available due to confidentiality concerns, but upon reasonable request, data will be shared by Prof Nazarius Mbona Tumwesigye, a co-author (naz@musph.ac.ug); stripped of original data identifiers so as to ensure confidentiality. Only variables used for the analysis will be shared.

## Abbreviations

AIC: Akaike Inclusion Criteria
CI: Confidence Interval
DEFF: Design Effect
DFID: Department for International Development
DHS: Demographic Health Survey
EA: Enumeration Area
ECI: Erreygers Concentration Index
FP: Family Planning
GLM: Generalized Linear Models
mCPR: Modern Contraceptive Prevalence Rate
MMR: Maternal Mortality Ratio
ODK: Open Data Kit
OR: Odds Ratio
OWC: Operation Wealth Creation
PMA: Performance Monitoring for Action
PR: Prevalence Ratio
RISE: Reducing High Fertility rates and Improving Sexual Reproductive Health Outcomes
SDG: Sustainable Development Goals
SAACO: Savings and Credit Cooperative Societies
TFR: Total Fertility Rate
UBOS: Uganda Bureau of Statistics
UDHS: Uganda Demographic Health Survey
UNCST: Uganda National Council for Science and Technology
UPE: Universal Primary Education
USE: Universal Secondary Education
VHT: Village Health Team

## Footnotes

1 Modern methods include: oral contraceptive pills, implants, injectables, contraceptive patch, vaginal ring, intrauterine device, female and male condoms, female and male sterilization, vaginal barrier methods (including the diaphragm, cervical cap and spermicidal agents), lactational amenorrhea method, emergency contraception pills, standard days method, basal body temperature method, Two-Day method and sympto-thermal method

2 For binary variables (*b*ℎ−*a*ℎ) equals one.
